# The Identification and Distribution of Cattle XCR1 and XCL1 among Peripheral Blood Cells: New Insights into the Design of Dendritic Cells Targeted Veterinary Vaccine

**DOI:** 10.1371/journal.pone.0170575

**Published:** 2017-01-27

**Authors:** Kun Li, Guoyan Wei, Yimei Cao, Dong Li, Pinghua Li, Jing Zhang, Huifang Bao, Yingli Chen, Yuanfang Fu, Pu Sun, Xingwen Bai, Xueqing Ma, Zengjun Lu, Zaixin Liu

**Affiliations:** State Key Laboratory of Veterinary Etiological Biology, Lanzhou Veterinary Research Institute, Chinese Academy of Agricultural Sciences (CAAS), Lanzhou, China; Katholieke Universiteit Leuven Rega Institute for Medical Research, BELGIUM

## Abstract

The chemokine (C motif) receptor 1 (XCR1) and its ligandXCL1 have been intensively studied in the mouse and human immune systems. Here, we determined the molecular characteristics of cattle XCR1 and XCL1 and their distribution among peripheral blood cells. Cattle XCR1 mRNA expression was mainly restricted to CD26^+^CADM1^+^CD205^+^MHCII^+^CD11b^-^ cells in blood that were otherwise lineage marker negative (lin^-^); these represented a subset of classic dendritic cells (DCs), not plasmacytoid DCs. Some of these DCs expressed CD11a, CD44, CD80 and CD86, but they did not express CD4, CD8, CD163 or CD172a. Cattle XCL1 was expressed in quiescent NK cells and in activated CD8^+^ T cells. Cattle XCR1^+^ DCs migrated chemotactically in response to mouse, but not to human, XCL1. The distribution characters of cattle XCR1 and XCL1 suggested a vital role in regulation of acquired immune responses and indicated a potential for a DC targeted veterinary vaccine in cattle using XCL1 fused antigens.

## Introduction

The chemokine (C motif) receptor 1 (XCR1) is a conserved marker on a subset of dendritic cells (DCs) with cross-presenting function in humans and mice[1, 2]. XCR1 was found to be selectively expressed on mouse CD8^+^ DCs[3], human CD141^+^ DCs [4] and pig CD172a^low/neg^CD11c^+^ DCs[5], by staining using specific antibody and XCL-mCherry vaccibody. These cross-presenting DCs are now termed XCR1^+^ DCs or cDC1. Currently, information about such cross-presenting DCs remains relatively rare for domestic animal species. Due to higher transcription of XCR1 and Clec9A genes, and superior efficacy for presentation of soluble antigen to CD8^+^ T cells, sheep lymph CD26^+^ DCs were proposed to be equivalents to mouse CD8^+^ DCs [6]. Furthermore, sheep CD26^+^CD172a^-^ DCs derived from upper aero-digestive tract were involved in self-antigen presentation and tolerance induction[7]. In cattle, the best described DCs are from afferent lymph and several teams have reported two subsets: a major CD172a^+^CD26^-^CD13^-^CD11a^-^CD1b^high^ subset and a minor CD172a^-^CD26^+^CD13^+^CD11a^+^CD1b^low^subset [8]. Cattle blood DCs were divided into three subsets: CD4^+^MHCII^-^lin^-^ pDCs, CD11c^+^MHC^+^lin^-^ cDCs and a novel CD11c^-^MHCII^+^lin^-^ cDCs [9]. In addition, differences in CD205 expression led to the division of the CD11c^+^MHCII^+^lin^-^ cDCs into two populations differing in TLR expression and T cell activation [10, 11]. However, cattle blood CD26^+^ DCs remain to be identified.

Comparative transcript data among mouse, human and sheep showed that DC subsets with cross presentation function highly express XCR1, C-type lectin-like receptor 9 member A (Clec9A), and cell adhesion molecule 1 (CADM1)[6, 12, 13]. Therefore, identification of these molecules distribution in cattle could allow identification of DC subsets with cross presentation function. Due to the rarity of XCR1^+^ DCs and lack of relevant antibodies for analysis of cattle cells, identification of DCs bearing the XCR1 receptor is a challenge. Here, we investigate the expression of XCR1 as well as its ligand XCL1 in cattle peripheral blood cells. Recent studies demonstrated that specific delivery of exogenous antigens to DCs via XCR1 efficiently enhances both cellular and humoral immunity responses [14–16]. Therefore, our identification of this DC subset in cattle will be useful for development of DC targeted vaccines against intracellular pathogens.

## Materials and Methods

### Ethics statement

All animal experiments were performed according to protocol by the Animal Ethics Procedures and Guidelines of the People's Republic of China, and the study was approved by the Animal Ethics Committee of LVRI, Chinese Academy of Agricultural Sciences (Permit No. LVRIAEC2016-008). The cattle were humanely bred and acclimated for at least one week before experimentation.

### Peripheral blood mononuclear cells (PBMCs) purification

Heparinized venous blood was sampled from the jugular vein of one year old Qinchuan cattle (Bostaurus), a Chinese breed of beef cattle. The cattle were clinically healthy, foot-and-mouth disease virus free and reared in clean livestock house. PBMCs were purified by density centrifugation using Hisopaque 1.083 (Sigma-Aldrich, USA) according to manufacturer instructions.

### Cloning of cattle XCR1 and XCL1

Total RNA from PBMCs was extracted using RNeasy Mini Kit (Qiagen, German) and digested with the RNase-Free DNase Set (Qiagen, German). Complementary DNA (cDNA) was produced by reverse transcription and used as a template for PCR reaction. The primers in Table 1 were designed basis on the predicted sequences under accession number XM_015459678(cattle XCR1) and NM_175716 (cattle XCL1) from Genbank in NCBI, and used for amplification of the coding regions of cattle XCR1 or XCL1 genes. The obtained fragments were ligated into pEASY-T1 vectors (Takara, Dalian, China) and sequenced in GENEWIZ NJ Laboratory (GENEWIZ, Nanjing, China). The deduced amino acid sequences for XCR1 or XCL1 of cattle were aligned with those of other species including horse, pig, monkey, mouse and human using the Clustal W program in DNA Star software (DNASTAR, Inc, USA). The TM helixes and extracellular domain of XCR1 and the secondary structures of XCL1 were predicted by Protein Prediction System (https://www.predictprotein.org/). The signal peptide cleavage sites of XCL1 from cattle and other species were predicted by SignalP 4.0 server.

**Table 1:** Primers used for amplification cattle XCR1 and XCL1 genes.

Gene	Primers (5’→3’)	^a^Ta (°C)	Expected products (bp)
**XCR1**	F:ATGGAGCCCTCAGACATCCCG	56.0	1002
	R: TCAATAGAAGGAGATGCCCTCG		
**XCL1**	F: ACTGCACAGCTCAGAGGGACCT	56.0	386
	R: TAGGGAAGGGACAAAGTGCTGG		

^a^ Annealing temperatures

### Isolation of blood cell populations

Monocytes were directly isolated from PBMCs using human CD14 MicroBeads (Miltenyi, Biotec). The isolation of other populations involved indirect magnetic separation via an LS column (Miltenyi, Biotec). The isolation of CD26^+^ DCs was performed in a two-step procedure: lineage negative (lin^-^) cells first were isolated by depletion with lineage antibodies(anti-bovine CD3/CD11b/CD14/CD21/CD335) using a MidiMACA separator via the LD column (Miltenyi, Biotec), thenCD26^+^ DCs were isolated by positive selection from the effluent via a MS column(Miltenyi, Biotec). For isolation of the lin^-^subsets, PBMCs were depleted by the LD column using the lineage antibodies in combination with selective one or two antibodies in Table 2, and the effluent cells were used as respective lin^-^subset population.

**Table 2:** Antibodies used to isolate cattle blood cell populations.

Antibody	Clone	Isotype^1^	Source
**CD3**	MM1A	IgG1	Monoclonal Antibody Center, Washington state University (MAC, WSU)
**WC1/WC1FITC**	CC101	IgG2a	AbDSerotec
**CD14**	CC-G33	IgG1	AbDSerotec
**CD21/CD21 RPE**	CC21	IgG1	AbDSerotec
**CD335/CD335 FITC**	AKS1	IgG1	AbDSerotec
**CD11b**	MM12A	IgG1	MAC, WSU
**CD11a**	HUH73A	IgG1	MAC, WSU
**CD11c**	BAQ153A	IgM	MAC, WSU
**CD44**	BAT31A	IgG1	MAC, WSU
**CD4/CD4 RPE**	CC8	IgG2a	AbDSerotec
**CD8 RPE**	CC63	IgG2a	AbDSerotec
**CD8a**	CACT80C	IgG1	MAC, WSU
**CD26**	CC69	IgG1	AbDSerotec
**CD44**	BAT31A	IgG1	MAC, WSU
**CD80**	ILA159	IgG1	MAC, WSU
**CD86**	ILA190A	IgG1	MAC, WSU
**CD163**	LND68A	IgG1	MAC, WSU
**CD172a**	CC149	IgG2b	AbDSerotec
**CD205**	CC98	IgG2b	AbDSerotec
**Anti-CADM1 mAb-Biotin**	3E1	Chicken IgY	MBL international

Isotype^1^: all the antibodies are derived of mouse host, exception for specific denotation.

### Cell stimulation

The purified cattle subset cells (1×10^6^ cells/ml) were seeded in 24-well plates with RPMI1640 media supplemented with 10% FBS, 25 mM HEPES, 50 μM β-mercaptoethanol and 100 μM penicillin-streptomycin. After one hour, cells were stimulated with PMA (50 ng/ml, Sigma-Aldrich, USA) and calcium ionomycin (1 μg/ml, Sigma-Aldrich, USA), or with concanavalin A (5 μg/ml, Sigma-Aldrich, USA) or with PBS buffer as control. Cells were collected after stimulation for 4 h, 8 h, 12 h, 16 h, 20 h and 24 h. All the collected cells were immediately stored at -70°C for later analysis.

### SYBR green real time RT-PCR

The expression of XCR1, XCL, Clec9A and GAPDH gene of cattle subset cells was quantified using the primers in Table 3 on an ABI Prism 7500 Real-Time PCR System (Applied Biosystems), with 100 ng cDNA as the starting template. These primers were designed basis on our cloned sequences of cattle XCR1 (KU641031), XCL1 (KU641032), Clec9A (KU641044) and GAPDH (NM_001034034) using Primer-Premier 5.0 (Premier Biosoft International, Palo Alto, CA, USA). SYBR green real time RT-PCR reaction system in total of 20 μl volume was prepared in accordance with SYBR Premix Ex TaqII (TaKaRa Bio, Dalian) instructions. Two-step amplification with an initial denaturation step of 95°C for 30 seconds, followed by 40 cycles of 95°C for 5 seconds and 61°C for 34 seconds, was performed to detect XCR1, XCL, Clec9A and GAPDH gene. The 2^-ΔΔCt^ method was used to evaluate gene expression of isolated cattle subset cells. The GAPDH gene was selected as internal control and PBMCs as calibrator sample, respectively.

**Table 3:** Primers used for SYBR green real-time RT-PCR.

Gene	Primers (5pecte	^a^Ta (°C)	Expected products (bp)
**XCR1**	F: TGCTGTGGGTCTTGGTGAA	61.0	100
	R: GGCAACAGGCAGGAGAACA		
**XCL1**	F: ACCATCAAGGAGGGCTCTGT	61.0	113
	R: CTGTCTATCTTTTGGACGGCTTTT		
**Clec9A**	F: TGGTGTCTTGTGATGGTGATCTTAT	61.0	175
	R: GGTTGGGGTTTCTCTTCCACTGT		
**GAPDH**	F: TCGGAGTGAACGGATTCG	61.0	227
	R: ATCTCGCTCCTGGAAGATG		

^a^ Annealing temperatures.

### Identification of cattle dendritic cell subsets by flow cytometry

Since DCs represented less than 1% total PBMCs[9], monocytes, T cells, B cells and NK cells and other lineage marker cells were first depleted using magnetic separation, then the lin^-^ cells, stained and analyzed by flow cytometry. The anti-bovine antibodies for staining are outlined in Table 4. Lin^-^ cells were first stained with anti-MHCII-PE (IgG2a), anti-CD11c (IgM), anti-CADM1 biotin and/or anti-CD4 FITC(IgG2a) /or CD205 FITC (IgG2b) /or anti-CD172a (IgG2b) for 30 minutes at 4°C, followed by staining with streptavidin APC-Cy7, rat anti-mouse IgG1 PE-CF594, anti-mouse IgM PE-Cy7 and/or anti-mouse IgG2b FITC. After blocking with mouse IgG (Thermo Fisher), anti-CD26 Alexa Flour647 was added and incubated for further 20 minutes at4°C. The dead cells were stained with Fixable Viability Stain 620 (BD Horizon^™^). The stained cells were fixed with 2% paraformaldehyde PBS buffer and analyzed by Becton Dickinson FACS verse (San Jose, CA, USA). Cell debris was excluded from analysis based on scatter signals.

**Table 4:** Primary and secondary antibodies used for 6-color flow cytometric analysis of cattle DCs subsets.

Antibody	Clone	Isotype^1^	Source
**CD3**	MM1A	IgG1	MAC, WSU
**CD14**	CC-G33	IgG1	AbDSertec
**CD21**	CC21	IgG1	AbDSertec
**CD335**	AKS1	IgG1	AbDSertec
**CD11b**	MM12A	IgG1	MAC, WSU
**CD11c**	BAQ153A	IgM	MAC, WSU
**MHCIIPE**	CC158	IgG2a	Abcam
**CD4 FITC**	CC8	IgG2a	AbDSertec
**CD26 Alexa Fluor647**	CC69	IgG1	AbDSertec
**CD172a**	CC149	IgG2b	AbDSertec
**CD205 FITC**	CC98	IgG2b	AbDSertec
**Anti-CADM1 mAb-Biotin**	3E1	Chicken IgY	MBL international
**Anti-mouse IgM PE-Cy7**	eB121-15F9	Rat IgG2a	eBioscience
**Anti-mouse IgG1 PE-CF594**	A85-1	Rat IgG1	BD Horizon^™^
**Anti-mouse IgG2b FITC**	R12-3	Rat IgG2a	BD Pharmingen^™^
**Streptavidin APC-Cy7**			BD Pharmingen^™^
**Fixable Viability Strain 620**			BD Horizon^™^

Isotype^1^: all the antibodies are derived of mouse host, exception for specific denotation.

### Cells migration assay

Cells migration assay was performed as described previously [2]. Migration of cattle CD26^+^ DCs was evaluated using Transwell insert polycarbonate membranes in 24-well plates (5 μm pore size; Corning). In brief, 1×10^5^ cells were suspended in 100 μl chemotaxis medium (RPMI 1640, 0.5%BSA) and seeded into the upper chamber. The lower chamber was filled with medium containing mouse or human XCL1 (R&D Systems) and the medium without XCL1 as blank control. The cells were incubated for 4 hours at 37°C with 5% CO_2_. The migratory cells in the low chamber were stained with Quick Count/Viability Reagent (GE Heathcare) for 5 min and analyzed by GE^™^ Cell image System. The percentage of migrated cells was calculated by the following formula: [number of migrated cells chemotaxised by XCL1- number of migrated cells in blank control]/[number of input cells] ×100. All experiments were independently repeated three times in three cattle and performed with triplicate wells each time.

## Results and Discussion

### Molecular characteristics of cattle XCR1 and XCL1

**Fig 1:**
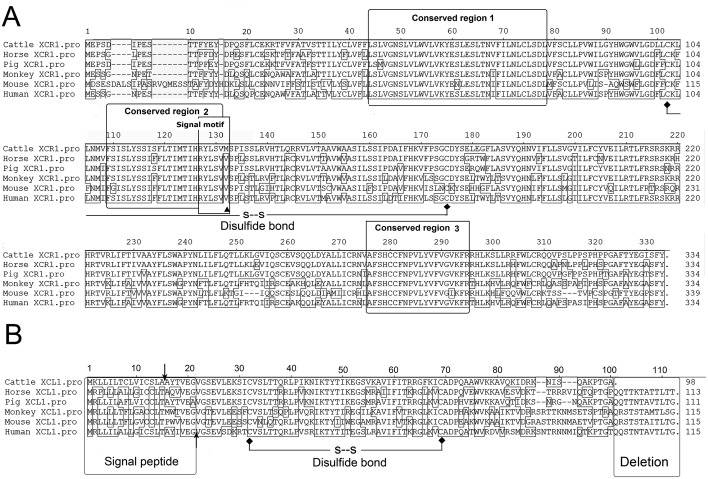
Alignment of cattle XCR1 and XCL1 deduced from the nucleotide sequence of the cloned cDNA. (A)The cloned cattle XCR1 showed 89, 92, 77, 71 and 78% aa identity homology with that of horse, pig, monkey, mouse and human, respectively. The conserved regions were marked with black frame. The mutation site in signal motif and Cystine sites forming potential disulfide were tagged in black circle and diamond patterns, separately. (B)The cloned cattle XCL1 showed 67, 78, 61, 57 and 67% aa identity homology with that of horse, pig, monkey, mouse and human, respectively. The different signal peptide cleavage sites were denoted with arrow.

The cloned cattle XCR1 and XCL1 were sequenced and deposited in GenBank under accession numbers KU641031 and KU641032. Alignment of cattle XCR1 with that of representative mammals (S1 Table) showed one potential disulfide bond (Cys102-Cys175) in all members and three conserved regions including region 1 (aa44-78), region 2 (aa109-131) and region 3 (aa276-295) (Fig 1A). Nevertheless, the signal motif “HRYLSV**V**”, which was conserved in the second intracellular loop of XCR1 in other species and involved with G protein coupling[17], occurred one amino acid mutant to “HRYLSV**M**” in the corresponding region of XCR1 in cattle, suggesting a possible difference in downstream signaling pathways of XCR1 between cattle with other species. The overall identity of cattle XCR1 with porcine, equine, human, rhesus monkey, and mouse XCR1 was 92, 88, 78, 77, and 71%, respectively.

The obtained coding region sequence of cattle XCL1 is 294 bp in length and encodes a secreted protein with 97 aa. Alignment of cattle XCL1 with that of representative mammals (S1 Table) showed that sequence difference in the N-terminal and deletion of 12 aa in the C-terminal region, although typical disulfide bond (Cys32-Cys69) existed in XCL1 of all the members (Fig 1B). The C-terminal deletion made cattle XCL1 a small size in length. However, this may not be involved with function alteration, as was revealed in chemokines of other families[16].

The signal peptide cleavage site of cattle XCL1 was predicted between Ala 15 and Ala 16, which resulted in a shorter signal with length of 15 aa, compared with cleavage sites between Gly 21 and Val 22 in all other XCL1 homologous (Fig 1B). These could indicate the difference in size of XCL1 mature proteins between cattle with other mammals.

### Cattle XCR1 is uniquely expressed on CD26^+^CADM1^+^CD205^+^CD11b^-^lin^-^ DCs

**Fig 2:**
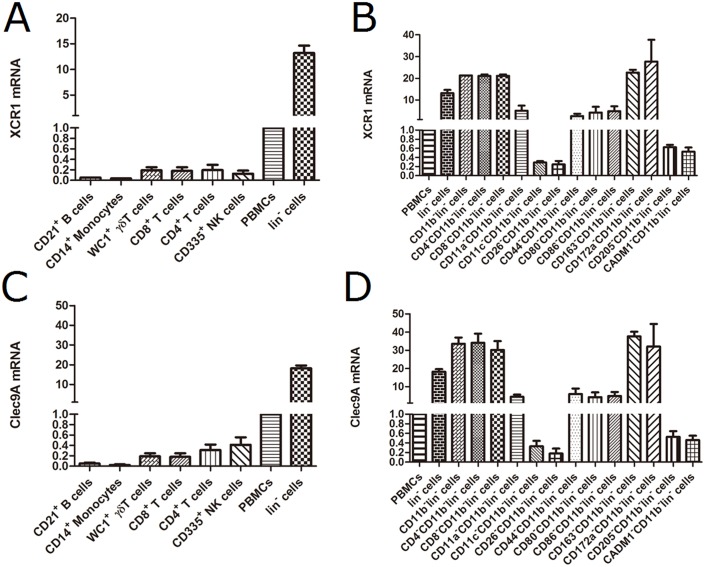
Analysis of gene expression of cattle XCR1 and Clec9A from isolated blood subset cells by RT-PCR. The relative expression of cattle XCR1 mRNA from isolated subset cells (A) and lin^-^ subset cells (B). The relative expression of cattle Clec9A mRNA from isolated subset cells(C) and lin^-^ subset cells (D). Data are representative of four independent experiments in three cattle. Error bars represent standard deviation.

Based on the RT-PCR results, XCR1 mRNA was nearly undetectable, and was below that of PBMCs, in monocytes, B cells, CD4^+^ T cells, CD8^+^ T cells, WC1^+^ γδ T cells, NK cells. By comparison, the mRNA of cattle XCR1 was obviously expressed within the lin^-^ cells, which gave a 15-fold higher signal than PBMCs (Fig 2A). To determine which cells expressed XCR1 mRNA, the lin^-^ cells were subjected to a series of antibody-dependent depletions of cells bearing specific markers (Fig 2B). Depletion of the major group of CD11b^+^ myeloid cells from the lin^-^ population increased the XCR1 mRNA signal, and this was further increased by depletion of cells bearing CD4, CD8, CD163 and CD172a. However, the XCR1 mRNA signal was lost from the CD11b^-^lin^-^ cells on further depletion of cells bearing CD11c, CD26, CD205 and CADM1. Thus, the XCR1 expressing cells were CD26^+^CADM1^+^CD205^+^CD11c^+^CD11b^-^CD4^-^CD8^-^CD163^-^lin^-^, apparently a DC subset.

In contrast to the clear results above, the CD11b^-^lin^-^ cells showed a partial reduction in XCR1 mRNA expression when depleted of CD11a, or CD80 or CD86. This suggests cattle XCR1^+^DCs represent a heterogeneous population with differences in activation state. In addition, the large variation in cattle XCR1 mRNA expression in CD172a^-^CD11b^-^lin^-^ cells may be related to the activation state or levels of this DC population.

### Cattle XCR1^+^cells represent a subset of conventional DCs not plasmacytoid DCs

**Fig 3:**
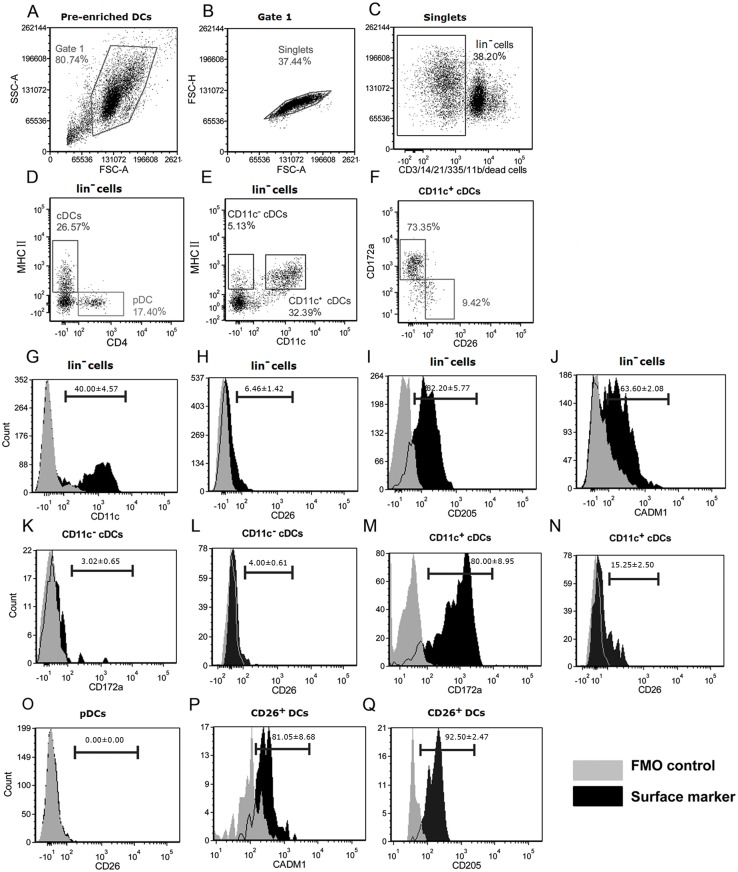
Phenotypic characterization of cattle blood DC subsets. 6-color flow cytometric was performed to determinate the distribution of the CD26, CADM1, CD205 and CD172a on subsets of cattle blood DCs. The pre-enriched DCs (A) obtained by depletion of lineage cells (anti-bovine CD3/CD11b/CD14/CD21/CD335) from PBMCs were subjected to flow cytometry. Gate 1 (B) was selected to exclude cells debris with lower values of SSC-A and FSC-A, and further analyzed to gate singlets based on diagonal streak of FSC-A and FSC-H plot. Both of dead cells and the remained lineage cells (C) were together excluded by staining with Fixable viability Stain 620 and anti-mouse IgG1 PE-CF594. lin^-^ cells were then gated to identify the MHCII^+^ and CD4^+^cells (D) as well as the MHCII^+^ and CD4^+^ cells (E). CD11c^+^ cDCs were further gated to analysis the plot (F) of CD26^+^ and CD172a^+^ cells. Overlap histograms for surface expression of CD11c (G), CD26 (H), CD205 (I) and CADM1 (J) on lin^-^ cells, CD172a (K) and CD26 (L) on CD11c^-^cDCs, CD172a (M) and CD26 (N) on CD11c^+^cDCs, CADM1 (P) and CD205 (Q) on CD26^+^ DCs, as well as CD26 (O) on pDCs, based on FMO control. Numbers in histograms represent average percentage of cells expressing the surface molecules in three cattle, and error bar indicate standard error.

In view of the limitations of the depletion approach, we sought to further define the potential XCR1^+^ DCs using the markers identified above in combination with markers representing cDCs and pDC by 6-color flow cytometry (Fig 3). Previous data on cattle suggested that amongst the lin^-^cells cDCs are CD4^-^MHC II^+^ while pDCs are CD4^+^MHCII^-^. Previous data on mice, humans and monkeys, as well as porcine blood, pointed to two populations of cDCs, CD172a^+^XCR1^-^ and CD172a^-^XCR1^+^[18–20]. The markers used above, in particular CD26, were correlated with this earlier information.

The flow cytometry data indicated that amongst the cattle blood lin^-^ cells the proportion expressing surface CD11c, CD205, CADM1 and CD26 was 40.0%, 82.2%, 63.6% and 6.5% respectively (n = 3; Fig 3G, 3H, 3I and 3J). Clearly there was overlap, and from the data above CD26 was the most selective marker of the XCR1 expressing cells. Further analysis indicated no CD26^+^ cells were among the CD4^+^MHCII^-^ gate. In conjunction with the data above, this indicates that cattle XCR1^+^ cells represented a subset of cDCs and not pDCs. Further analysis showed that cattle blood cDCs consisted of CD26^+^CD172a^-^ and CD26^-^CD172a^+^ subsets, representing 15.3% and 80.0% of cDCs respectively (n = 3; Fig 3M and 3N).

Overall combining this data from flow cytometry with the XCR1 expression on depleted populations, the XCR1 expressing population of cattle blood represents the CD26^+^CD172a^-^ subset of cDCs. This correlates closely with the XCR1 expressing cDC1 population of other species.

### Clec9A expression correlates with XCR1 expression in cattle

Clec9A mRNA was detected in CD11b^-^lin^-^ cells bearing CD11c, CD26, CD205and CADM1 (Fig 2C and 2D). Thus, Clec9A shares a similar expression pattern toXCR1 in cattle. In contrast, pigs were reported not to express Clec9A[5]. In mice, Clec9A is expressed by pDCs as well as cDCs, and the latter also express CD8[21]. These expression patterns revealed that cattle are like humans, not mice, in the co-incident expression of Clec9A and XCR1. Given this, using antibody against Clec9A would be an alternative way of targeting the XCR1^+^ DC population in cattle. Indeed, targeting vaccine antigens to cDC1 via Clec9A antibody induced efficient cytotoxic T cells effectors and high antibody responses in mice model[22], as well as potential antitumor immunity in human[23]. Thus, delivery vaccines via Clec9A antibody to XCR1^+^ DC in cattle could also be a promising approach for obtaining protective antibody and cytotoxic T cell immune responses to pathogenic agents.

### Cattle XCL1 is expressed by quiescent NK cells and by activated CD8^+^ T cells

**Fig 4:**
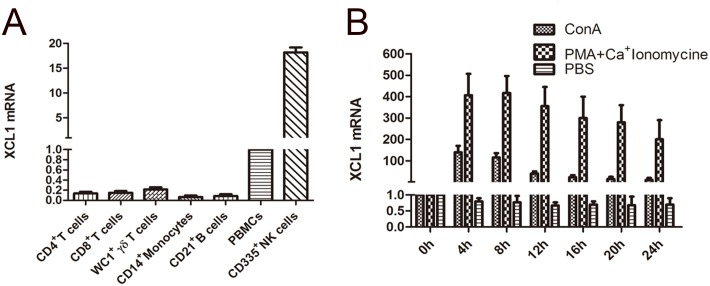
Analysis of gene expression of cattle XCL1 in isolated blood subset cells and CD8^+^ T cells after stimulation. The relative expression of cattle XCL1 mRNA from isolated subset cells(A) as well as obtained CD8^+^ T cells after stimulation with the PMA plus calcium ionomycin, or the Concanavalin A, respectively for different time (B). All the data above was representative of four independent experiments in three cattle.

The expression by cattle blood cells of the ligand for XCR1, XCL1, was also assessed by RT-PCR. Under steady state conditions cattle XCL1 was exclusively expressed in CD335^+^ NK cells and not in other cells in prior to activation (Fig 4A). To assess the potential for XCL1 gene expression, the purified cattle blood cell subsets were stimulated with PMA and ionomycin for different times. Cattle XCL1 mRNA in CD8^+^ T cells was extremely increased (up to 400-fold) to the peak levels at 8 h (Fig 4B). This marked increase in XCL1 mRNA was not observed in other cattle subsets cells after stimulation. The expression of XCL1 mRNA by CD8^+^ T cells was also increased after stimulation with concanavalin A, although the extent was lower than with PMA and ionomycin. These cell distribution characteristics of XCL1 and XCR1 suggested that NK cells naturally and CD8^+^ T cells after activation could interact with CD26^+^ DCs via the XCL1-XCR1 axis in the cattle immune system.

### Cattle XCR1^+^ DCs are attracted by mouse XCL1

**Fig 5:**
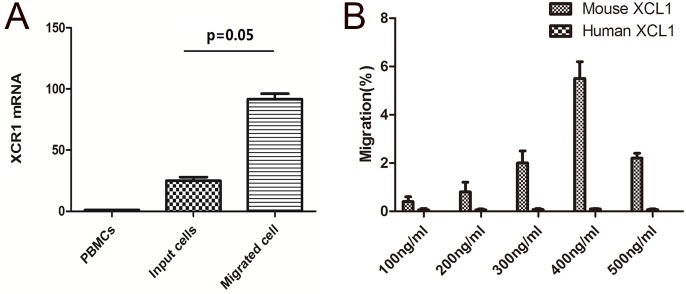
Chemotactic migration of cattle XCR1^+^ DC by mouse XCL1 and human XCL1. (A) The relative expression of cattle XCR1 mRNA from input cells (CD26^+^ DCs) in upper chamber and migrated cells (XCR1^+^ DC) in lower chamber. Difference in XCR1 expression between the two populations was calculated using one-tailed nonparametric t-test (Mann-Whitney test). A significant difference was defined as *P* ≤ 0.05. (B) Transwell assay on CD26^+^ DCs by mouse XCL1 and human XCL1 with concentration varied from 100 ng/ml to 500 ng/ml.

To ensure that the XCR1 mRNA expression we measured led to a functional outcome, the XCR1 expressing DCs were tested in migration assays. Mouse XCL1 induced the migration of CD26^+^lin^-^ DCs, not CD26^-^lin^-^ DCs, inducing a maximum migration of 5% of the input cells (Fig 5). The relative cattle XCR1 gene expression of the migrated cells in the lower chamber was significantly higher than that of input cells remaining in the upper chamber (p = 0.05;Fig 5A), indicating the migrated cells selected for the XCR1^+^ DCs in the input CD26^+^lin^-^ DCs. However, although cattle XCL1 had a slightly higher sequence identity with human XCL1 than mouse XCL1, no migration of either CD26^+^lin^-^ DCs or of CD26^-^lin^-^ DCs were observed when using human XCL1 at different concentrations (Fig 5B).

Given to the difference in XCL1 sequences between cattle and mouse, we speculate that the function domain involved the interaction of cattle XCL1 with XCR1 could be located in the middle region, not at each end of XCL1. In contrast, a previous study showed that the native N-terminal sequence of human XCL1 was necessary for receptor activation[24]. Thus, it seems likely that human XCL1 binds and activates XCR1 in a manner different from mouse and cattle XCL1.

More recently, targeting XCR1^+^ DCs using XCL1 fused antigens had induced potent CD8^+^ T cell cytotoxicity in a mouse model and human[14]. Thus, these data suggest the XCR1 activity in cattle blood CD26^+^ DCs allows it to interact with its ligand XCL1, which could be used as a marker for targeting XCR1^+^ DCs and exploring of DCs targeted molecule.

### Summary

Our cloning of cattle XCR1 demonstrated that it is a highly conserved molecule across species. XCR1 is expressed on CD26^+^CADM-1^+^CD205^+^CD11b^-^lin^-^ cattle blood cells, representing a subset of cDCs, not pDCs. XCR1 expression correlates with Clec9A expression in cattle. This defines a XCR1^+^ cDC1 population in cattle. Cattle XCL1 is expressed by quiescent NK cells and by activated CD8^+^T cells. The XCL1 of mouse, not of humans, had chemotactic activity on cattle XCR1^+^DCs. These results point to two ways of targeting vaccine antigens to the XCR1 population in cattle, via the XCL1 or via antibodies against Clec9A.

## Supporting Information

S1 Table Data sources of XCR1 and XCL1 used for alignment. (DOCX)

S1 File Data of cattle XCR1 (sheet 1) and Clec9A (sheet 2) mRNA expression in blood cells populations and lin^-^ subsets. (XLSX)

S2 File Data of cattle XCL1 mRNA expression in blood cells populations (sheet 1) and CD8^+^T cells (sheet 2) stimulated by PMA, Con A and PBS for 0h, 4h, 8h, 12h, 16h, 20h and 24h. (XLSX)

S3 File Data of cattle XCR1 mRNA expression in input cells (CD26^+^lin^-^ DCs) and migrated cells (XCR1^+^ DCs). (XLSX)

S4 File Data of migrated percentage of cattle XCR1^+^ DCs attracted by mouse XCL1 and human XCL1 with concentration varied from 100 ng/ml to 500 ng/ml. (XLSX)
