# Supplementary material for: The Identification and Distribution of Cattle XCR1 and XCL1 among Peripheral Blood Cells: New Insights into the Design of Dendritic Cells Targeted Veterinary Vaccine
Source: PLoS One. 2017 Jan 27;12(1):e0170575. doi: 10.1371/journal.pone.0170575 (PMC5271332; doi:10.1371/journal.pone.0170575)
Supplement: S1 Table — (DOCX) [file pone.0170575.s001.docx]

**S1 Table. Data sources of XCR1 and XCL1 used for Alignment.**

| Organism | Breed | Country | Gene name | Accession number | Length (aa) |
| --- | --- | --- | --- | --- | --- |
| Cattle | Qinchuan | China | XCR1  XCL1 | KU641031  KU641032 | 333  97 |
| Mouse | Mus musculus  C57BL/6 | - | XCR1  XCL1 | NM_011798  NM_008510 | 338  114 |
| Human | Homo sapiens | - | XCR1  XCL1 | NM_005283  NM_002995 | 333  114 |
| Pig | Domestic pig | China | XCR1  XCL1 | AB119265  EU743945 | 333  110 |
| Horse | Equus caballus ferus Boddaert (tarpan) | - | XCR1  XCL1 | XM_005600757  XM_001490960 | 333  112 |
| Monkey | Macaca mulatta | USA | XCR1  XCL1 | XM_001114298  NM_001032947 | 333  114 |

“-” denotes sources unavailable.
